# Immune Escape Mechanisms in Colorectal Cancer Pathogenesis and Liver Metastasis

**DOI:** 10.1155/2014/686879

**Published:** 2014-01-16

**Authors:** Massimo Pancione, Guido Giordano, Andrea Remo, Antonio Febbraro, Lina Sabatino, Erminia Manfrin, Michele Ceccarelli, Vittorio Colantuoni

**Affiliations:** ^1^Department of Sciences and Technologies, University of Sannio, 82100 Benevento, Italy; ^2^Medical Oncology Unit, Fatebenefratelli Hospital, 82100 Benevento, Italy; ^3^Department of Pathology “Mater Salutis” Hospital, 37045 Legnago (VR), Italy; ^4^Department of Surgery and Oncology, University of Verona, 37129 Verona, Italy; ^5^Bioinformatics Lab, BIOGEM scrl, 83031 Ariano Irpino (AV), Italy

## Abstract

Over the past decade, growing evidence indicates that the tumor microenvironment (TME) contributes with genomic/epigenomic aberrations of malignant cells to enhance cancer cells survival, invasion, and dissemination. Many factors, produced or *de novo* synthesized by immune, stromal, or malignant cells, acting in a paracrine and autocrine fashion, remodel TME and the adaptive immune response culminating in metastasis. Taking into account the recent accomplishments in the field of immune oncology and using metastatic colorectal cancer (mCRC) as a model, we propose that the evasion of the immune surveillance and metastatic spread can be achieved through a number of mechanisms that include (a) intrinsic plasticity and adaptability of immune and malignant cells to paracrine and autocrine stimuli or genotoxic stresses; (b) alteration of positional schemes of myeloid-lineage cells, produced by factors controlling the balance between tumour-suppressing and tumour-promoting activities; (c) acquisition by cancer cells of aberrant immune-phenotypic traits (NT5E/CD73, CD68, and CD163) that enhance the interactions among TME components through the production of immune-suppressive mediators. These properties may represent the driving force of metastatic progression and thus clinically exploitable for cancer prevention and therapy. In this review we summarize results and suggest new hypotheses that favour the growing impact of tumor-infiltrating immune cells on tumour progression, metastasis, and therapy resistance.

## 1. Introduction

More than 1.2 million colorectal cancers (CRCs) are diagnosed every year worldwide, accounting for approximately 10% of all cancers [1, 2]. The death rate from CRC has been dropping for more than 20 years, mostly due to earlier screenings and improved treatments. In spite of this, CRC remains the fourth most common cause of cancer-related death in western countries [1–5]. Recent evidence suggests that accumulation of genetic and epigenetic alterations in malignant colonic cells progresses through at least three distinct pathways: chromosomal instability (CIN), microsatellite instability (MSI), and CpG island methylator phenotype (CIMP) [6–8]. CIN is the most common type of genomic instability occurring in 60%–80% of CRCs and results in an imbalance of the chromosome number “manifested as aneuploidy.” MSI is an alternative pathway, accounting for 15–20% of sporadic CRCs in which the characteristic signature is deletion of repetitive regions of DNA that in most cases generates frameshift mutations in the coding sequences of genes leading to their inactivation. CIMP is a novel molecular instability pathway characterized by the widespread hypermethylation of CpG islands at several genomic loci [6–8]. Up to date, massive genomic studies have discovered *ERBB2* and *IGF2* amplifications as novel potential therapeutic targets; extensive molecular profiling studies have identified clinically and molecularly distinct subtypes of CRC [9–11]. In addition to cancer genome abnormalities, also the formation of an inflammatory microenvironment plays a pivotal role in CRC development and progression [12, 13]. CRC survival is highly dependent on the tumour stage at the time of diagnosis; over one-third of patients die within five years from the initial diagnosis and most of fatal outcomes result from liver metastases [14–17]. The metastatic process is a multistep event that entails cancer cells to escape from the primary tumour, survive in the circulation, seed at distant sites, and grow [14, 15]. It is well established that the metastatic spread is promoted by communications between tumour and immune cells via the secretion of cytokines, growth factors, and proteases that remodel the tumour microenvironment (TME) [12, 13]. Consistent with this idea, driver gene mutations (*APC*, *TP53*, *SMAD4*, *PIK3CA*, and *KRAS*) along with genomic and epigenomic instability determine tumour initiation, while the interaction of cancer cells with microenvironmental stimuli provided by nontransformed cells is needed to evolve towards a metastatic cancer [15–19]. The underlying molecular mechanism by which cancer cells acquire the ability to escape the primary tumour site, evade immune system eradication, and reestablish a new order is currently under intense investigation [19–23]. A number of studies have shown that infiltration and density of immunologic cells within primary tumours are mostly associated with patients' prognosis and sensitivity to therapy [19–23]. In particular, tumour associated-macrophages (TAMs), regulatory T cells (Treg), and the so-called purinergic signaling cascade are considered attractive targets for antitumour interventions [19–26]. This field is drawing increasing attention to identify new pharmaceutical targets and improve therapies' efficacy. In this review, we present results and suggest new hypotheses that underscore the growing impact of tumour-infiltrating immune cells in CRC progression. Furthermore, we discuss the advances in our understanding as to how the reciprocal communications between cancer and stromal cells impact liver metastasis formation and response to the therapy.

## 2. Infiltrating Immune Cells and CRC Progression

**Figure 1:**
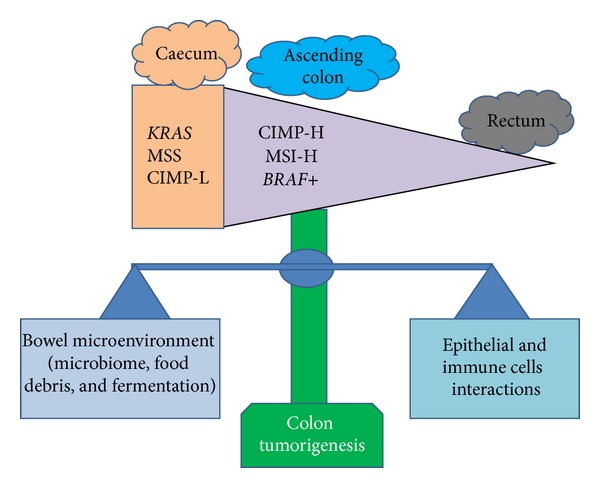
Bowel microenvironment influences CRC molecular features and progression. The CIMP-high, MSI-high, and *BRAF* mutations frequencies gradually increase from the rectum to ascending colon. Caecal cancers seem to represent a distinct subtype characterized by a higher frequency of *KRAS* mutations, a MSS, and CIMP-L phenotype. In the bowel microenvironment, changes in the balance between (1) microbiome, food debris, and bacterial fermentation products and (2) interactions with host cells (epithelial and immune cells) might predispose colon epithelial cells to certain molecular insults and differentially influence tumour development according to molecular features in preneoplastic cells. CpG island methylator phenotype: CIMP; microsatellite instability: MSI; CIMP-low: CIMP-L.

Most of cancer hallmarks are sustained to varying degree by genetic and epigenetic modifications of colon cancer cells and by stromal and immune cell types that contribute to generate a specific TME. A recent model proposed by Yamauchi et al. suggests that the CRC molecular features gradually change along bowel subsites and factors such as the interactions with the gut microbiota, biochemical components, innate immune system, and epithelial cells might trigger the initiating molecular events or, alternatively, influence tumour microenvironment to promote neoplastic progression [27] (Figure 1). CRC and other solid tumours contain infiltrates of diverse leukocyte subsets including both myeloid- and lymphoid lineages that do not conform to the classical picture of an inflammatory immune response. The evidence provided is particularly robust for tumour infiltrating T lymphocytes (TILs) or tumour associated macrophages (TAMs) suggesting that both adaptive and innate antitumour immune responses play key roles in cancer progression [28]. By contrast, the potential role in human cancers of short-lived innate immunity cells, such as dendritic cells or tumor-associated polymorphonuclear neutrophils (TAN), has received poor attention. However, recent studies have revealed the existence of potential neutrophil subsets in CRC patients showing a tumor-promoting (N2) or tumor-inhibiting (N1) phenotype in response to the transforming growth factor-*β* (TGF-*β*) [29, 30]. In addition, evidence supports the ability of TAN to express cytokine, chemokine-encoding genes, and a wider array of growth factors such as tumour-necrosis factor (TNF), hepatocyte growth factor (HGF), and vascular endothelial growth factor (VEGF), suggesting a potential role in tumor progression and angiogenesis [28–30]. A striking finding is that an elevated neutrophils blood count in either tumour or blood has a prognostic significance in several neoplasms. Accordingly, also the neutrophil/lymphocyte ratio has been associated with poor clinical outcome in CRC [30]. The significance of TAN in human cancers remains to be fully clarified and needs further experimental confirmation.

## 3. T-Cell Signature and Immune Escape in CRC

Tumour tissues infiltration by immune cells, particularly those of the lymphoid-lineage, has extensively been studied and associated with the destruction of tumour cells, reduction of the tumour burden, and improved clinical prognosis [31]. Unfortunately, the underlying rationale of these observations remains elusive. Functional studies have established the existence of a tight interplay between genetic instability of tumour cells and the degree of antitumour immune responses mediated mainly by CD8+ T cells, also referred to as cytotoxic T cells (CTLs) [20, 31–33]. Moreover, the local immune infiltration appears to be closely associated with the different genetic instability signature (MSI and CIN) [32, 33]. CRCs with high levels of MSI (MSI-H+ CRC) are characterized by a strong local immune reaction, mainly by peritumoural lymphoid nodules (Crohn's-like reaction) and a dense infiltration of TILs, part of which are “activated and/or cytotoxic” [20, 31–38]. CIN+ CRCs, instead, exhibit reduced expression of cytotoxic T-cell markers and intratumoural density of Foxp3-positive regulatory T cells (Treg cells) [38–43].

These observations would explain why MSI-H+ CRCs tend to be more immunogenic than CIN+ CRCs and suggest that a dense infiltration of CTLs within TME could effectively mediate tumour cells destruction by releasing their lytic components [39–46]. Several studies have shown that MSI-H+ CRCs are associated with a better prognosis than CIN+ CRCs. Conversely, our own data indicate that some MSI-H+ CRCs are extremely aggressive and characterized by a reduced infiltration of “TILs,” suggesting that the mechanism underlying immune surveillance is only partially dependent on the MSI signature [46, 47]. The higher immunogenicity associated with MSI-H+ CRCs is currently explained by frameshift mutations in coding microsatellites that would render tumour cells vulnerable to recognition and attack by the host's immune system. In agreement, truncating mutations affecting the genes coding for human leukocyte antigen (HLA) class I antigen components have been identified as the major mechanism mediating HLA antigen presentation impairment in MSI-H+ CRC that has been found in about 30–60% of the lesions [48, 49]. Antigen presentation abrogation by HLA class I antigens, therefore, represents a potent mechanism of immune evasion that protects tumour cells from the attack by cytotoxic T cells. Concomitantly, mismatch repair (MMR) deficiency provokes a strong local and systemic antitumoural immune response, the so-called “Th1-response” due to the generation of frameshift peptide antigens or novel tumour-specific antigens. This suggests that HLA class I alterations, while offering protection against local antitumoural immune responses, might interfere with the ability to form distant metastases. Whether MSI-H+ CRCs infiltrating T cells are recruited from the periphery or locally expanded due to cytokines or chemokines generated by tumour cells remains unknown at present.

## 4. Tumour Associated Macrophages and CRC Progression

**Figure 2:**
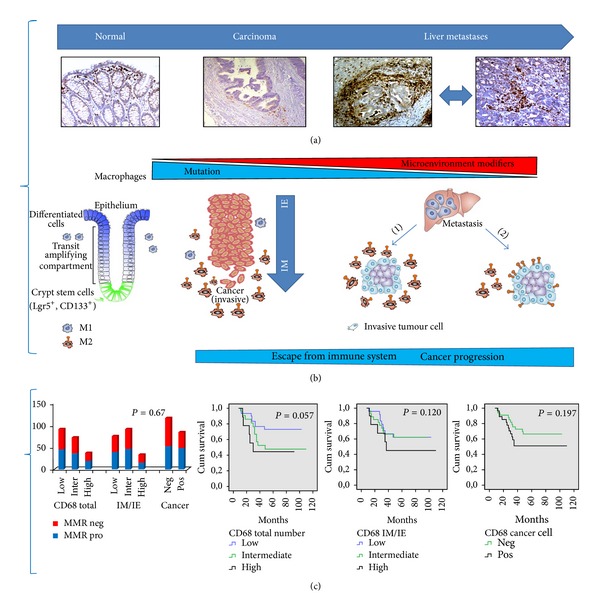
Recruitment of tumour-promoting immune cells contributes to progression and metastasis. (a) Representative images of tissue sections (normal colonic mucosa, carcinoma, and liver metastases) immunostained with CD68, a marker of macrophages with a M2-phenotype. (b) A schematic and simplified model as to how TAMs might contribute to tumour development and metastasis. CRC arises by genetic damages, mutations, and deregulated signaling pathways; metastatic spread, instead, is promoted by communications between tumour and immune cells. Normal colonic tissues show specialized macrophages residing in well-defined niches. The functions of TAMs during tumour progression may depend on their intrinsic plasticity and adaptability mediated by factors controlling the balance between the M1 and M2 phenotype. We suggest that at least two mechanisms may play a role in the prometastatic function of TAMs: (1) M2-macrophages produce a dense layer surrounding invasive cancer cells resulting in heterotypic interactions between the tumour and the host stroma; (2) invasive cancer cells can acquire immunophenotypic traits (i.e., CD68) due to cell fusion between macrophages and cancer cells facilitating homotypic interactions with the host stroma and TAMs. (c) Polarized, CD68 positive, M2-macrophages may have distinct functions depending on their density or distribution between the invasive edges and intraepithelial areas (ratio IM/IE ×100). CD68 expression is not confined to the infiltrating TAMs but is extended to a significant fraction of the malignant cells. These parameters correlate with the mismatch repair (MMR) status and patients' disease specific survival in our cohort of 82 CRC patients. IE: intraepithelial; IM: invasive margin; MMR pro and MMR neg indicate MMR proficient and deficient tumours, respectively. The *P* value is reported in each graph.

Macrophages are one of the most common nontumour cell types present in the TME and play a central role in inflammation and tumour development. The significance of macrophages in the metastatic process has recently been emphasized and drawn a great deal of interest [50–54]. Tumour infiltrating macrophages, also indicated as TAMs (Figure 2(a)), derive from circulating monocytic precursors and are recruited to tumour sites by several molecules, such as the chemokines CCL2 and CCL5, VEGF, TGF-*β*, and colony stimulating factors (GM-CSF and M-CSF) [53, 55]. Tumours also recruit a variety of immature myeloid cells, often referred to as myeloid-derived suppressor cells (MDSCs), thought to create a permissive environment for subsequent invasion and tumour growth [56–58]. Interestingly, recent findings have shown that mononuclear MDSCs can further mature into macrophages [53–57]. At least two TAMs phenotypes contribute to cancer initiation and promotion: M1 proinflammatory macrophages have pronounced antitumour activity being cytotoxic to tumour cells. Persistence of an inflammatory response, however, can be detrimental and help cancer initiation and/or progression through the generation of mutation-inducing reactive oxygen species and nitrogen-free radicals. Macrophages can be subverted to a tumour-promoting M2 phenotype in response to immune-suppressive cytokines secreted by tumour tissues that can foster metastasis through extracellular matrix remodeling, angiogenesis, and suppression of antitumour immune responses (Figure 2(a)) [58–62]. M-CSF, PGE2, TGF-*β*, IL-6, and IL-10 have the potential to modulate and polarize monocytes mainly into M2 macrophages by influencing fundamental aspects of CRC biology [57–62]. The protumoural effects of TAMs are due to the fact that they synthesize a large array of growth factors including epidermal growth factors (EGF), TGF-*β*, VEGF, and several proteolytic enzymes such as matrix metalloproteinases (MMPs) that degrade ECM proteins. These factors, in turn, promote tumor expansion, neoangiogenesis switch, motility, and invasion (Figure 1(a)). Recent evidence suggests that TAMs are educated to perform tasks that enhance metastasis through the construction of a premetastatic niche that represents a new environment favourable to seeding and growth of tumour cells (Figure 1(b)) [52–58]. Macrophages density in CRC has also been linked with patients' prognosis; although a considerable body of data has been produced, their role in colorectal cancer is still controversial [61–65].

## 5. Polarized TAMs and Development of Liver Metastasis

Taking into account the recent trends in cancer immunology, we report here some representative examples of the roles that TAMs play in fostering metastasis and maintaining evasion of the immune surveillance. We propose that the many TAMs distinct functions during tumour progression may depend on their intrinsic adaptability to positional schemes obtained by factors controlling the balance between tumour-suppressing and tumour-promoting activities (Figures 2(a) and 2(b)) [60, 66]. In normal conditions, the interplay between colonic cells and host immune system is preserved by a highly structured organization with specialized macrophages residing in well-defined niches to fulfill their physiological functions. In primary tumours, either oncogenic alterations or changes in TME establish a new equilibrium that can be further modified during metastatic progression. We suggest that at least two mechanisms underlie the TAMs prometastatic functions: (1) M2-macrophages can form a dense barrier around invasive cancer cells resulting in heterotypic interactions between tumour cells and the surrounding stroma that compromise the integrity of the host tissues; (2) invasive cancer cells can acquire immunophenotypic traits, for example, due to fusion of macrophages with cancer cells, which facilitate homotypic interactions between the host stroma and TAMs (Figure 2(b)). Collectively, these observations suggest that TAMs protumour activity is associated with distinct mechanisms that are independent of the MSI signature, as suggested by our own data (Figure 2(c)). Accordingly, an increased M1-macrophages infiltration at the tumour front has recently been correlated with a better prognosis in CRC patients [65]. The antitumour effects of TAMs could then potentially be due to the presence of a significant number of M1-polarized macrophages, able to mediate killing of tumour cells in peritumoural areas. In contrast, a density of M2-macrophages at the invasive front higher than in intraepithelial regions of the tumour leads to enhanced invasion through secretion of chemokinetic growth factors that remodel the extracellular matrix, thus shortening patients' survival (Figures 2(b) and 2(c)). In conclusion, still little is known about the macrophage subtypes and their associated molecular profiles in cancer. Given that multiple subpopulations of TAMs exist within a tumour, a promising field of research is focused on questioning as to how the phenotypic equilibrium temporally and spatially changes over the course of tumour progression.

## 6. The Evolution of the Cancer Niche and Immune Suppressive Pathways

According to the linear progression model of tumour development, genetic damages, mutations, and deregulated signaling pathways establish the initiation step. Subsequently, the immune cell populations that infiltrate the tumour mass exert a primary suppressive role; however, these immune cells, due to their intrinsic plasticity, can undergo phenotypic changes that enhance tumour cell dissemination and metastasis depending on the presence of accessory stromal cells, the local cytokine milieu, and tumour-specific interactions (Figure 2(a)). Several lines of evidence suggest that MDSCs establish cell clusters called “premetastatic niche” that precede the arrival of even a single metastatic tumour cell at distant sites; whether MDSCs have a direct role in enhancing the metastatic process is only at the beginning to be elucidated [66–68]. In this perspective, it has been suggested that the properties of an “invasive niche” are already established within the primary tumour, where cancer cells, macrophages, and endothelial cells establish a special setting in which paracrine signaling loops lead to increased intravasation and dissemination of cancer cells [66–73]. Accordingly, the ability to metastatic dissemination could be viewed as the evolution of the cancer niche in which (1) interactions of a genetically initiated cancer cell with specific host cells facilitate survival and a malignant behavior; (2) inflammation-driven phenotypic plasticity alters the antigenic landscape of tumour cells; (3) secreted factors, such as chemokines, cytokines, and exosomes, generate a “microenvironment” that remodels local tissues by promoting malignant phenotypes and immune escape. Intriguingly, the recently proposed mechanisms to explain both metastatic niche evolution and tumour immune escape include (1) malignant cells that acquire functional and phenotypic characteristics of immune cells by expressing the macrophage scavenger receptors CD163 or CD68; (2) aberrant activation of oncogenic pathways in cancer cells which facilitates the interaction with stromal cells; (3) production of anti-inflammatory cytokines and immunosuppressive metabolites (adenosine) that ultimately generates a poorly immunogenic TME [26, 68–73].

## 7. Novel Immune Suppressive Pathways and Metastatic Evolution: A Key Role of NT5E/CD73

**Figure 3:**
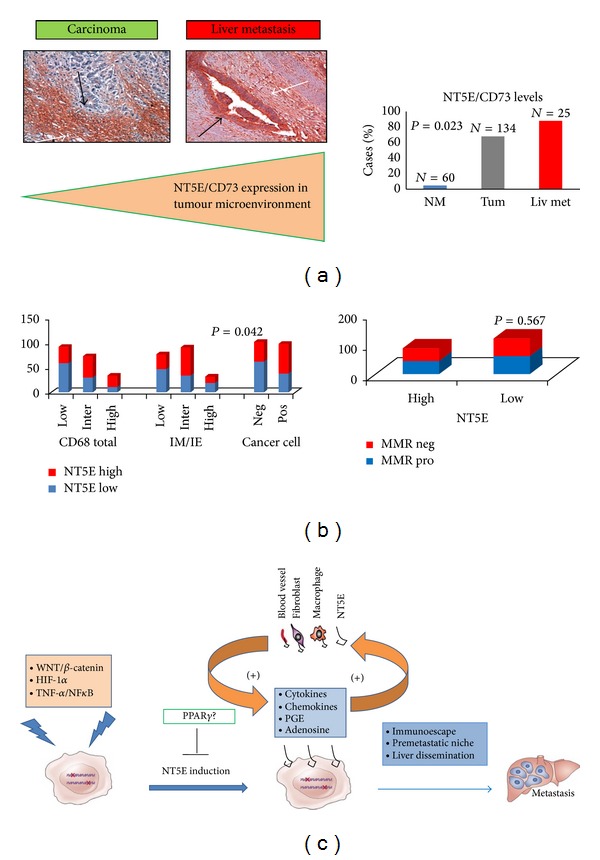
NT5E/CD73 facilitates escape from immune-surveillance and orchestrates the tumour-stroma interplay to promote cancer growth and metastasis. (a) Representative images of NT5E/CD73 immunostaining in a primary carcinoma and corresponding liver metastasis. White and black arrows indicate the immunostaining in the stromal compartment and malignant colonic cells, respectively, magnification 10X. (b) NT5E/CD73 immunostaining is higher in liver metastatic tissues than in primary tumours or normal colonic mucosa and significantly correlates with CD68 infiltration. NT5E/CD73 expression is not correlated with the MMR status in our cohort of CRC patients. (c) Schematic drawing of the proposed mechanism(s) involved in metastasis-promoting actions of NT5E/CD73. In physiological conditions, NT5E/CD73 hydrolyzes AMP to adenosine, an important mediator that binds A2A receptors on activated CD4 effector T-cells, decreasing their proliferation and cytokine production, hence mediating immunosuppressive effects. NT5E/CD73 is expressed in a variety of stromal cells (macrophages, endothelial cells, B-lymphocytes, and Treg-cells). A variety of oncogenic pathways can induce ectopic NT5E/CD73 expression in CRC cells, enhancing paracrine/autocrine interactions (activation of A2B receptors) between malignant colonic, hematopoietic, and nonhematopoietic cells to sustain immunosurveillance escape and cancer cell migration. *PPARγ* (peroxisome proliferator-activated receptor gamma) has been suggested as a possible NT5E/CD73 antagonist through still unknown mechanisms. IE: intraepithelial; IM: invasive margin; Liv Met: liver metastases; MMR pro and MMR neg indicate MMR proficient and deficient tumours, respectively. The *P* value is reported in each graph.

Among the factors capable of evading immune surveillance and altering the tumour cell antigenic landscape, NT5E/CD73 has recently received great attention [74–78]. NT5E/CD73 encodes a GPI-anchored cell surface enzyme abundantly expressed in hematopoietic and endothelial cells that converts ATP to adenosine, a potent immunosuppressor. Adenosine exerts its tumour-promoting effects in a paracrine and autocrine fashion by activating its cognate receptors (the adenosine receptors) expressed by tumour, endothelial, or immune cells [76–78]. Interestingly, NT5E/CD73 is the rate-limiting enzyme in the production of extracellular adenosine, thus, representing a checkpoint in the conversion of proinflammatory ATP into immunosuppressive adenosine [76–78]. By applying a new bioinformatic approach, we recently identified NT5E/CD73 as a novel CRC prognostic biomarker [79]. Elevated NT5E/CD73 levels in either malignant epithelial cells or TME strongly correlate with poor patients' outcome [79]. Consistently, NT5E/CD73 expression is higher in liver metastasis than in primary tumour or normal mucosa and is significantly linked with TAMs expression profile but not with the MMR status (Figures 3(a) and 3(b)). Our findings and those reported in the literature suggest a schematic and simplified model in which NT5E/CD73 metastasis-promoting actions appear to be the result of a close cooperation between cancer, stromal, and inflammatory cells (Figure 3(c)). A variety of protumorigenic pathways (hypoxia-inducible factor (HIF-1*α*), Wnt/*β*-catenin, and TNF-*α*/NF*κ*B) can synergize to induce *NT5E/CD73* expression in malignant cells enhancing paracrine/autocrine interactions with malignant colonic, hematopoietic, and nonhematopoietic cells to sustain immune surveillance evasion, premetastatic-niche evolution, and cancer cell migration. Only limited information is available about *NT5E/CD73* downregulation; we recently suggested PPAR*γ* (peroxisome proliferator-activated receptor *γ*) as a possible *NT5E/CD73* repressor [79]. All together these results suggest that multiple mechanisms affect *NT5E/CD73* expression in cancer and stromal cells (endothelial cells, fibroblasts, and TAM) contributing to the evolution of metastatic niches and evasion of immune surveillance (Figure 3(c)). Further studies and new model systems are required to study the dynamic changes occurring in stromal cells that enable initiated tumour cells to survive and progress towards metastasis.

## 8. Tumour Microenvironment Plasticity as a Determinant of the Response to Therapy

Over the past decade, the scenario of metastatic CRC (mCRC) treatment has deeply changed. The benefits of classic anticancer agents have been strenghtened by novel, target-oriented molecules whose efficacy, in combination with standard chemotherapy, has been demonstrated [80–84]. An emerging concept in anticancer therapy involves the mobilization of several types of MDSCs following treatment with traditional or targeted therapies that may contribute either to a lack of response or to an acquired drug resistance [84–88]. Although it is still unknown how MDSCs mobilization occurs, it has been demonstrated that MDSCs precursors are recruited to metastases by VEGFA signaling through VEGFR2 [84, 85].

Endothelial cells, among the stromal cell types, are crucial to metastatic dissemination and outgrowth; accordingly, drugs able to target these cells are the most advanced in clinical trials [84–89]. A VEGF antagonist, commercially known as “Bevacizumab,” increases survival in patients with mCRC when combined with chemotherapy, in particular with fluoropyrimidines, Irinotecan, and Oxaliplatin [84]. Recently, a wide range of new molecules targeting angiogenesis have been tested in clinical trials. Interestingly, two drugs, “Aflibercept”, a VEGF and PIGFs-antagonist, and “Regorafenib”, a multi-Tyrosine Kinase Inhibitor, have significantly improved the progression free survival in a Phase III randomized trial [89–92].

**Figure 4:**
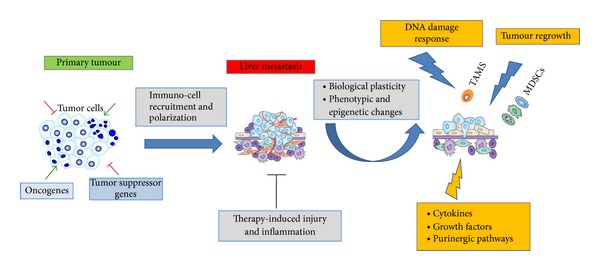
Tumour and immune cells plasticity in therapy-induced tumour tissue damage and development of therapy resistance. Immune cells polarization, soluble mediators, and extracellular matrix components of the tumour microenvironment contribute to progression and liver metastasis by constructing the so-called “premetastatic niche.” Therapy-induced tumour tissue injuries lead to interconnected phenotypic alterations of tumour and immune cells. Activity of several drugs (5-FU; gemcitabine and other cytotoxic agents) on (the) malignant cells can be associated with the concomitant TAMs and MDSCs activation or expansion due to enhanced polarization and phenotypic plasticity in metastatic tissues. Consistent with this hypothetical model, extensive tumour tissue injuries and necrosis activate the “inflammasome” and enhance the release of cytokines (IL-1*β*) and growth factors (TGF-*β*) and likely activate immune-suppressive pathways mediated by adenosine production (purinergic pathways). In addition, tumour tissue damage, following cytoreductive therapy, results in epigenetic alterations, DNA damage response, increased expression of drug transporters, and DNA-repair enzymes in malignant cells which contribute to protect against cytotoxic drugs and result in therapy resistance and tumour relapse.

Therapeutic blockade of macrophage recruitment or chemokine signalling has recently been shown to improve survival after chemotherapy [88–94]. Accordingly, the anticancer agent “Trabectedin,” employed in the treatment of soft tissue sarcomas, selectively depletes TAMs *in vivo* [95]. TAMs or MDSCs, in contrast, can activate the “inflammasome” through the release of cathepsin B and IL1*β* in response to 5-FU, a mechanism that reduces the anticancer activities of this drug [85]. The effects of other classic cytotoxic agents such as Oxaliplatin and Irinotecan on TAMs remain to be elucidated. Furthermore, anti-EGFR therapy activates M2-macrophages or MDSCs, resulting in release of immunosuppressive and tumour-promoting mediators (Figure 4) [85, 96]. According to these data, irradiated macrophages produce signals that include CD95 ligand, TNF*α*, nitric oxide, and superoxide, suggesting a model based on the intrinsic adaptability of immune and cancer cells in response to a genotoxic therapy [87, 88, 97]. Anticancer drugs can determine rapid or slow interactions among TME components promoting DNA damages or epigenetic modifications, which result in increased expression of drug transporters, DNA-repair enzymes, and chromosomal instability genes, all mechanisms leading to tumour regrowth (Figure 4). In spite of the translational promise of the targeted therapy, our understanding of the relationships between mechanisms of resistance and TME remodeling remains very limited. This reinforces the importance of a full comprehension of the intricacy of cell interactions occurring in TME.

## 9. Immunogenic Cell Death and the Purinergic Signalling in Cancer Therapy

Collectively, MDSCs can either enhance or antagonize the antitumour efficacy of cytotoxic chemotherapy, cancer-cell targeting antibodies, and immuno therapeutic agents depending on the treatment and tumour type. Therapy resistance has been linked with a new concept defined as “immunogenic cell death” (ICD) [24, 26], an event mainly mediated by damage-associated molecular patterns (DAMPs). DAMPs are either secreted or released (such as ATP); some become enriched or are *de novo* exposed on the outer leaflet of the plasma membrane (such as calreticulin (CRT) and heat shock protein 90 (HSP90). This mechanism is characteristic of dying, stressed, or injured cells and these molecules can act as either adjuvant or danger signals for the innate immune system [24, 26]. Studies have shown their interactions with phagocytosis, purinergic and pattern-recognition receptors (PRRs), are required for ICD that ultimately leads to the activation of a potent anticancer immunity. In this context, accumulating data underscore the therapeutic potential for targeting the adenosinergic system [26, 76, 77], in particular, the key molecule NT5E/CD73. Numerous studies in a variety of murine tumour models have also highlighted that loss of NT5E/CD73 function can efficiently delay tumour growth and confer metastasis resistance [76, 77, 79]. In the TME, a shift towards ATP accumulation might be crucial in mediating an effective antitumour response; thus, depletion of NT5E/CD73 may be clinically relevant as an ICD inducer, supporting the notion that the adenosinergic system is a relevant target in cancer. Blocking NT5E/CD73 signaling could have two important effects: to rescue the endogenous adaptive antitumour immune responses and to inhibit the metastatic potential of tumour cells. Therefore, investigations aimed at elucidating the mechanisms underlying the signalling activated by the adenosinergic system-ICD interactions not only will improve our understanding of this important process but will also contribute to the development of new strategies for cancer therapy.

## 10. Concluding Remarks

Genetic and epigenetic alterations are the key driver events in the initial transformation of normal colonocytes; growing lines of evidence suggest that the stromal and inflammatory cells within the tumour microenvironment and the circulation play a fundamental role in metastatic dissemination. In fact, a large series of factors, routinely synthesized or *de novo* expressed by the microenvironment during tumour development, act in paracrine and autocrine fashion and induce immunosuppression, immune-mediated tumour progression achieving a new metastable order. On the basis of the various trends developed over the past decade in the cancer immune surveillance field, cancer inflammation, and cancer therapy, we suggest a list of biologic properties that are crucial to modify the tumour microenvironment and educate malignant, stromal, and inflammatory cells towards a metastatic phenotype: (1) the intrinsic plasticity of immune cells in response to paracrine and autocrine signals and adaptability to novel and adverse environmental conditions (i.e., response to genotoxic stimuli); (2) alterations of positional schemes by factors controlling the balance between tumour-suppressing and tumour-promoting activities (i.e., evolution of a premetastatic niche); (3) acquisition of immune-phenotypic traits (NT5E/CD73, CD68, and CD163) by cancer cells that enhance the interactions with TME components through the production of immune-suppressive mediators. If confirmed, this working hypothesis could be used to stratify patients carrying defined genetic lesions and identify molecular profiles of TME subtypes in cancer biopsies before, during, and after therapy. The mechanisms regulating TME functions in normal conditions and in response to therapy, however, are still far to be completely understood, especially for the possibility to exploit such information in the clinical management of patients and development of new strategies of cancer therapy.
